# Postmenopausal estrogen receptor positive breast cancer and obesity associated gene variants

**DOI:** 10.17179/excli2020-2860

**Published:** 2021-07-05

**Authors:** Asuman Özgöz, Fadime Mutlu Içduygu, Aysegül Yükseltürk, Hale Samli, Kuyas Hekimler Öztürk, Zuhal Baskan, Ilknur Tütüncü

**Affiliations:** 1 Kastamonu School of Medicine, Department of Medical Genetics, Kastamonu University, Kastamonu, Turkey; 2 School of Medicine, Department of Medical Genetics, Giresun University, Giresun, Turkey; 3 Fazil Boyner Faculty of Health Sciences, Department of Nutrition and Dietetics, Kastamonu University, Kastamonu, Turkey; 4 School of Veterinary Medicine, Department of Genetics, Uludag University, Bursa, Turkey; 5 School of Medicine, Department of Medical Genetics, Süleyman Demirel University, Isparta, Turkey; 6 Department of Medical Oncology, Acibadem Bursa Hospital, 16110 Bursa, Turkey

**Keywords:** ADIPOQ, FTO, polymorphism, postmenopausal, breast cancer

## Abstract

Obesity is one of the most important health risks in postmenopausal women. Molecular pathways that are connected with obesity are believed to interact with the pathogenesis of breast cancer (BC). The aim of this research was to study the polymorphisms of two obesity-associated genes ADIPOQ and FTO that are also related to the pathogenesis of BC. Obesity-associated gene polymorphisms ADIPOQ rs1501299 and rs2241766, and FTO rs1477196, rs7206790, rs8047395, and rs9939609 were studied in 101 Turkish postmenopausal estrogen receptor-positive BC patients and 100 healthy control individuals. ADIPOQ rs1501299 was detected to be associated with protection against BC. The ADIPOQ rs1501299 TT genotype, the rs2241766 GT genotype and the G allele were found to be significantly higher in the control group. In addition, ADIPOQ rs1501299 polymorphism was protective in the recessive model and rs2241766 polymorphism was protective in the dominant model. While none of the FTO gene polymorphisms were found to be associated with BC, the frequencies of rs9939609 A allele and rs7206790 G allele were correlated with body mass index (BMI) in BC patients. ADIPOQ rs1501299 TT genotype, rs2241766 GT genotype, and G allele might be protective against BC in the Turkish population but this conclusion needs to be further verified.

## Introduction

Obesity is known to be one of the most important health risks worldwide and it is also associated with increased postmenopausal breast cancer (BC) risk (La Vecchia et al., 2011[20]; Pasha et al., 2019[30]). The molecular mechanism related to the connection between BC and obesity is unclear (Pasha et al., 2019[30]). BC patients have been reported to be mostly obese at diagnosis and, differently from in other cancers, to significantly gain weight after diagnosis. This situation has been demonstrated to be correlated with poor BC prognosis, decreased survival rates, and chemoresistance (Reddy et al., 2013[31]). The estrogen level in postmenopausal obese women tends to be 50-100 % greater than in women of normal weight. This leads them to have twice the risk of developing BC by triggering pathological situations leading to the disease (da Cunha et al., 2013[9]). High volumes of adipose tissue give rise to high estrogen levels, increased levels of cytokines including TNF-a, IL-6, and prostaglandin, insulin resistance, increased insulin-like growth factor activity, and oxidative stress; these are all reported to be leading causes of cancer formation (Akbari et al., 2018[1]). These factors contribute to the specific molecular pathways related to the progression of cell cycle; regulation of protein synthesis, and apoptosis (Lorincz and Sukumar, 2006[22]). Adiponectin (ADIPOQ), fat mass and obesity associated-protein (FTO), leptin, leptin receptor, serum paraoxonase/arylesterase, and melanocortin receptor-4 are specific obesity-associated genes, which, when they have defects, have also been reported to be associated with BC risk (da Cunha et al., 2013[9]; Vona-Davis et al., 2007[37]; Stephenson and Rose, 2003[35]).

Adipose tissue secretes many adipokines that act bioactively. ADIPOQ is the most frequently secreted adipokine and a decreased ADIPOQ level is demonstrated to be related to cancer progression (Stephenson and Rose 2003[35]; Ye et al., 2014[39]; An et al., 2012[4]).

The ADIPOQ gene, which is localized on 3q27, is expressed mainly in adipose tissue and its polymorphisms are associated with cancer risk by possibly affecting the protein levels in plasma (Pasha et al., 2019[30]). Decreased adiponectin levels were verified to increase BC, colorectal cancer and adenoma risk in two meta-analyses (Ye et al., 2014[39]; An et al., 2012[4]). In subgroup analysis performed in another meta-analysis, low adiponectin levels significantly correlated with breast, colon, endometrial, prostate, gastroesophageal cancer risks (Wei et al., 2016[38]). Adiponectin was found to prevent tumor development and decrease angiogenesis (Bråkenhielm et al., 2004[7]).

The FTO gene was the first reported obesity-associated gene in a genome-wide association study (GWAS) conducted in a Caucasian population. FTO polymorphisms were further verified to be associated with the risk of obesity in different populations (Kang et al., 2017[16]). Additionally, most obesity-related polymorphisms of the FTO gene are intronic and in strong linkage disequilibrium; for this reason, it has not yet been possible to determine SNPs affecting gene expression. However, the “A” risk allele of FTO rs9939609 SNP has been demonstrated to have the effect of increased expression of the gene (Berulava and Horsthemke, 2010[6]).

The FTO gene, mutations of which may conclude in serious growth retardation, increased metabolic rate and hyperphagia, is localized on 16q12.2 (Zeng et al., 2015[40]). Variants of the FTO gene have been reported to be associated with endometrial, pancreatic and breast cancer risk but not to be associated with prostate cancer risk. FTO triggers cancer risk via obesity-related carcinogenesis but the real molecular mechanism has not been elucidated (Chen and Du, 2019[8]). Although the functional importance of FTO variants has not been clarified, risk alleles are thought to affect epigenetic changes such as methylation (Zeng et al., 2015[40]). 

In the light of the above literature, the current study aimed to investigate the obesity-associated gene polymorphisms ADIPOQ rs1501299 and rs2241766, and FTO rs1477196, rs7206790, rs8047395 and rs9939609 in Turkish postmenopausal estrogen-receptor (ER) positive (+) BC patients and healthy control individuals in order to determine the links between them.

## Materials and Methods

101 post-menopausal ER (+) BC cases from the Department of Medical Oncology, Acıbadem Bursa Hospital (mean age: 62 ± 7.8 years), and 100 postmenopausal healthy individuals as a control (mean age: 52.6 ± 3.7 years), and who had all signed informed consent forms, were included in the research. Case information forms with detailed information were filled in for the patients and the control-group individuals. The Medical Research Ethics Committee of Acıbadem Mehmet Ali Aydınlar University approved the research project. 

DNA samples were isolated using a High Pure PCR Template Preparation Kit (Roche, Diagnostics GmbH, Mannheim, Germany) in accordance with the manufacturer's instructions. For genotyping, matrix-assisted laser desorption/ionization mass spectrometry with time-of-flight measurement (MALDI-TOF) was carried out after multiplex PCR on a Sequenom MassARRAY 4 analyzer, and mass spectra analysis was performed using the MassARRAY TYPER 4.0 software (Agena Bioscience, San Diego, USA), as previously described (Özgöz et al., 2019[28]).

The SPSS 18.0 software (SPSS Inc. Released 2009. PASW Statistics for Windows, Version 18.0. Chicago: SPSS Inc.) was used for statistical analyses. To evaluate the Hardy-Weinberg equilibrium, the chi-square test was used. BC patients and the control individuals' genotype and allele frequencies were compared using Pearson's chi-square and Fisher Exact tests. Logistic regression analyses were carried out and the Odds Ratio (OR) was calculated with 95 % confidence intervals (CIs). Demographic and clinical data were compared using the Student's t- and Mann-Whitney U tests. 

## Results

The clinical characteristics and demographic properties of the cases are shown in the demographic profile table (Table 1(Tab. 1)). In terms of the differences between the BC patients and the control individuals, age (p=0.000), age at first delivery (p=0.000), and family history (p=0.006) were found to be statistically significant.

The genotype and allele frequencies of ADIPOQ (rs1501299, rs2241766) and FTO (rs1477196, rs7206790, rs8047395, rs9939609) polymorphisms in the BC patients and control individuals are presented in Table 2(Tab. 2). The ADIPOQ rs1501299 TT genotype, the rs2241766 GT genotype and the G allele were found to be higher in the control group than in the BC case group (respectively, p=0.019, p=0.017and p=0.049). None of the differences in the frequency of the FTO (rs1477196, rs7206790, rs8047395, rs9939609) alleles and genotype between the groups were found to be statistically significant (all: p > 0.05). All the ADIPOQ and FTO SNPs were in Hardy-Weinberg equilibrium in both the BC case and the control groups.

BMI, Progesterone (PR), Her2/neu, metastasis, lymph node status, tumor grade, family history of BC patients and the studied SNPs of ADIPOQ were not related (all: p > 0.05). The FTO rs7206790 CG genotype and the rs9939609 TA genotype were correlated with increased BMI in BC patients (respectively, p=0.031 and p=0.005) (Table 3(Tab. 3)). 

## Discussion

Obesity has been reported to be correlated in a complex way with the risk of BC, as well as with the clinical outcomes of the ongoing disease (Jafari Nedooshan et al., 2017[11]). In postmenopausal women, one of the known risk factors for BC is obesity and up to 50 % of elderly women are thought to be obese (Simone et al., 2016[33]). However, the risk of BC in obese women seems to be affected by ethnicity and hormone receptor status: increased BMI has been reported to be correlated with BC risk especially in populations of Asian-Pacific origins and in ER (+) and PR (+) cases (John et al., 2013[12]). The risk of BC in obese women has been reported to be increased in relation to estrogen levels, inflammation, insulin metabolism, and molecules of blood originating in intracellular pathways. Measuring these factors has been suggested as a way of understanding the increased activation of intracellular signaling pathways related to BC risk. It is thought that evaluating these factors together with BC and obesity-associated gene polymorphisms will provide important information for improving diagnostic and prognostic procedures in order to treat obese BC patients (Simone et al., 2016[33]).

In a study by Kaklamani et al., two functional single nucleotide polymorphisms (SNPs) (rs1501299 and rs2241766) in the obesity-associated gene ADIPOQ were found to be related to BC for the first time in the literature. Some genotypes of these polymorphisms were associated with decreased BC risk (rs2241766 TG and GG), while some were associated with increased BC risk (rs1501299 TG and GG) (Kaklamani et al., 2008[15]). While some studies have reported associations, in particular of rs1501299 and BC risk (Pasha et al., 2019[30]; Macías-Gómez et al., 2019[23]; Méndez-Hernández et al., 2017[24]; Kaklamani et al., 2013[14]), some have failed to report an association (Slattery et al., 2015[34]; Nyante et al., 2011[27]). To the best of our knowledge there is no meta-analysis in the literature related to ADIPOQ in obese BC.

The current study is the first to evaluate ADIPOQ rs1501299 and rs2241766 SNPs in Turkish postmenopausal ER (+) BC patients. In this study, there were found to be fewer individuals with ADIPOQ rs1501299 TT genotypes (p=0.019) and rs2241766 GT (p=0.017) genotypes among the BC patients and it can therefore be suggested that this situation led to the Turkish population being better protected against BC.

In a study by Karaduman et al., performed with Turkish BC patients and controls, ADIPOQ levels in fresh-frozen breast tissue samples were measured using the ELISA method. In the study, increased ADIPOQ levels were correlated with BC risk in postmenopausal Turkish women (p=0.003), unlike in the literature. In addition, clinicopathological parameters such as BMI, hormone receptor status, lymph node involvement, tumor size and grade were not associated with increased ADIPOQ levels in breast tumor samples (p > 0.05). Karaduman et al. suggested that this result may have been due to tissues being studied, rather than blood samples (Karaduman et al., 2007[17]). However, we suggest that this disparity may not only have been related to the sample type (tissue versus blood), but also to ethnicity. Although the current study did not examine ADIPOQ levels, increased ADIPOQ levels have been reported in the literature to be neither associated with ADIPOQ SNPs nor with BC risk; our results are thus in line with those of Karaduman et al. In Erbay et al.'s study conducted in a Turkish population, rs1501299 and rs2241766 SNPs were reported not to be associated with BC risk (p > 0.05). The current study is also in line with Erbay et al.'s study as no association was found between these SNPs and BC risk in a Turkish population (Erbay et al., 2016[10]). However, our study differs from theirs as significant protective effects of some genotypes of these SNPs against BC were detected. In addition, none of the SNPs was correlated with clinopathological parameters in the current study (all: p > 0.05).

In contrast with the current study, other studies have found the following genotypes to be associated with BC risk: in a Kuwaiti population the rs1501299 GG genotype (also depending on BMI) and rs2241766; in a South Indian population the rs1501299 GT and rs2241766 TG genotypes (Al Khaldi et al., 2011[3]; Mohan Reddy et al., 2012[25]); in a Northeast Indian population the rs1501299 GT and rs2241766 TG genotypes (Khandouzi et al., 2016[18]); in an Egyptian population the rs2241766 TG and GG genotypes (Pasha et al., 2019[30]) in African-Americans the rs1501299 GT/GG genotypes (Kaklamani et al., 2013[14]).

No associations were reported between BC risk and ADIPOQ polymorphisms in Hispanics and White Americans (Kaklamani et al., 2013[14]; Teras et al., 2009[36]), but in the current study, with a different population, ADIPOQ rs1501299 was significantly protective against BC (p=0.010). In addition, the numbers of rs1501299 TT and rs2241766 GT genotypes and G alleles were found to be higher in the control group than the BC case group (respectively, p=0.019, p=0.017 and p=0.049), which demonstrates these genotypes and this allele may be protective against BC in our population. In the study, ADIPOQ rs1501299 SNP was also protective in the recessive model (TT VS GT+GG: OR=0.24, 95 % CI=0.08-0.67, p=0.004) and rs2241766 SNP was protective in the dominant model (TT VS GT+GG: OR= 0.50, 95 % CI=0.27-0.91, p=0.022) in BC. In a meta-analysis conducted in 4268 cancer cases and 6299 controls, ADIPOQ rs1501299 T allele was reported to be correlated with decreased cancer risk in Asian and Caucasian populations and the association was significant in the dominant model (GG vs. TT/GT, OR= 0.84, 95 % confidence interval [CI]: 0.77-0.92) (Li et al., 2014[21]). In another meta-analysis by Zhou et al., including 4936 cancer cases and 5432 controls, the ADIPOQ rs2241766 G allele was associated with decreased cancer risk in line with the current study, even though our study is only related to BC (Zhou et al., 2013[43]). In a study conducted in the Mexican population, again in line with the current study, there were found to be a higher number of rs2241766 GT (p=0.022) genotypes and G alleles (p=0.002) in the control group than in the BC case group and this SNP was suggested to be protective against ductal infiltrating breast cancer (DIBC) formation (Macías-Gómez et al., 2019[23]).

While the variations of the obesity-associated gene FTO were reported to be associated with BC risk in some populations (Zeng et al., 2015[40]; Chen et al., 2019[8]; Sadim et al., 2017[32]; Zhao et al., 2016[42]; Zhang et al., 2014[41]), they could not be associated with some others (da Cunha et al., 2013[9]; Jafari Nedooshan et al., 2017[11]; Mojaver et al., 2015[26]).

In the current study, the FTO gene, rs1477196, rs7206790, rs8047395, and rs9939609 SNPs were not associated with BC in Turkish postmenopausal ER (+) BC patients (all p > 0.05). In the literature there are conflicting results about the association of FTO gene SNPs with BC. In line with the current study the following SNPs were not associated with BC risk: in an Iranian population rs9939609 and rs1477196 (Mojaver et al., 2015[26]); in a Brazilian population rs9939609 (da Cunha et al., 2013[9]); in a Polish population rs9939609 (Kusinska et al., 2012[19]), and in a Kazakhstani population rs1477196 (Akilzhanova et al., 2013[2]). In a Chinese population, the FTO rs1477196 AA genotype was associated with lower BC risk in women with BMI < 24 kg/m^2^ (Zeng et al., 2015[40]), while, in a large scale study of those with European ancestry, rs9939609 was associated with BC risk (Zhao et al., 2016[42]). In a study by Kaklamani et al. conducted with Caucasian people from the USA, rs7206790, rs8047395, rs9939609 and rs1477196 were significantly associated with BC risk (Kaklamani et al., 2011[13]).

In the current study, interestingly, the FTO rs7206790 and rs9939609 SNPs significantly correlated with BMI in BC patients; the frequency of the rs9939609 A allele and rs7206790 G allele in BC patients with BMI ≥ 30 kg/m^2^ was found to be increased (respectively, p=0.005 and p=0.031). In particular, rs9939609 has been reported to be associated with increased BMI and related conditions, and it has been suggested that the A allele could be used to predict high BMI (Baručija-Özçoban et al., 2018[5]). The results of the current study are in line with this assertion, in terms of the presence of increased rs9939609 A allele in BC patients with raised BMI. It can be suggested that this SNP may increase disease risk related to BMI. None of the remaining clinopathological parameters were associated with FTO SNPs and BC risk (all: p > 0.05).

In conclusion, in the present study, we evaluated obesity-associated gene polymorphisms, namely, ADIPOQ rs1501299 and rs2241766, FTO rs1477196, rs7206790, rs8047395, and rs9939609 for the first time together in Turkish postmenopausal ER (+) BC patients. The numbers of ADIPOQ rs1501299 TT, rs2241766 GT genotypes and rs2241766 G allele were found to be significantly lower in BC patients compared to the controls. It is possible that these genotypes and this allele protect the Turkish population from BC, but this needs to be verified by future studies conducted of this population. None of the studied SNPs of FTO gene were found to be associated with BC risk in the current study, and they were also not associated with any of the clinopathological parameters, except with BMI which was correlated with the frequencies of the rs9939609 A allele and rs7206790 G allele in BC patients. 

If the supporting evidence about the possible effect of population-specific genomic alterations in cancer risk is taken into account (Park et al., 2018[29]), in our opinion, ADIPOQ rs1501299 in particular, which was significantly protective against BC in the present study, should be included in cancer evaluations and predictive biomarker panels designed for the Turkish population, together with other susceptibility and protective variants.

## Notes

Dr. Ilknur Tütüncü died on 22 September 2019.

## Funding

This study was funded by the Scientific Research Projects Management Coordination Office of Kastamonu University with the code number KÜBAP-01/2013-30.

## Conflict of interest

The authors declare that they have no conflict of interest.

## Authors' contributions

Blood and data collection for the cases in the study was performed by Zuhal Başkan; laboratory studies and data analysis were performed by Asuman Özgöz, Fadime Mutlu İçduygu, Ayşegül Yükseltürk, Hale Şamlı, Kuyaş Hekimler Öztürk. İlknur Tütüncü helped with data analysis. Asuman Özgöz and Fadime Mutlu İçduygu wrote the manuscript. All authors read and approved the final version of the manuscript except for İlknur Tütüncü.

## Acknowledgements

We are deeply sad to say that one of the authors, Dr. İlknur Tütüncü, died on 22 September 2019. We are very grateful to Prof. Dr. Ahmet Dursun, who read and contributed to the manuscript on behalf of Dr. Tütüncü.

## Figures and Tables

**Table 1:**
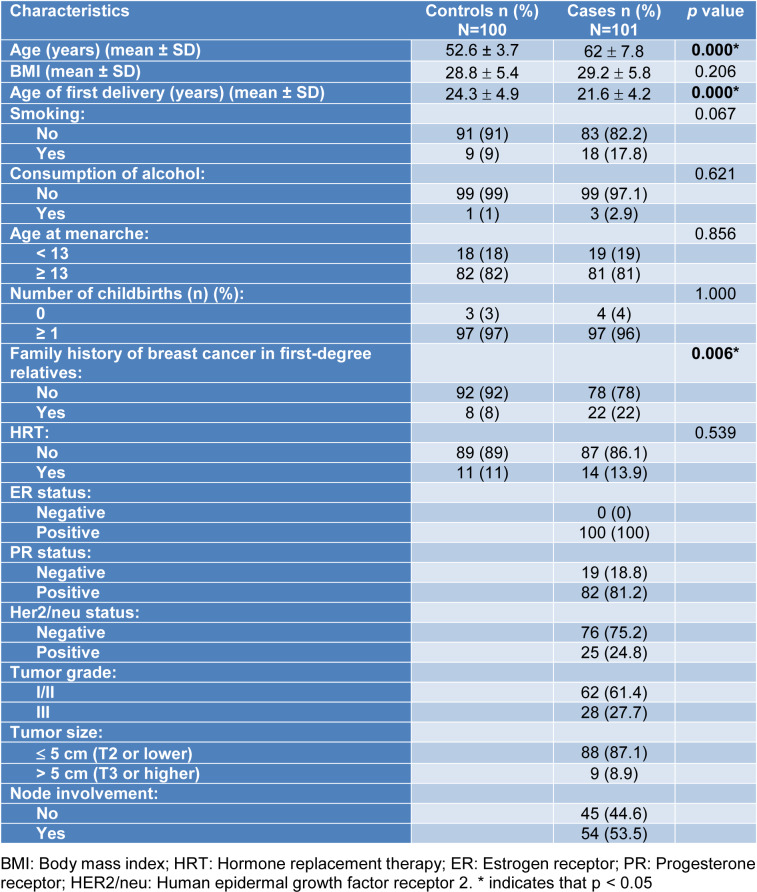
BC cases and the control group individuals' demographic and clinical characteristics

**Table 2:**
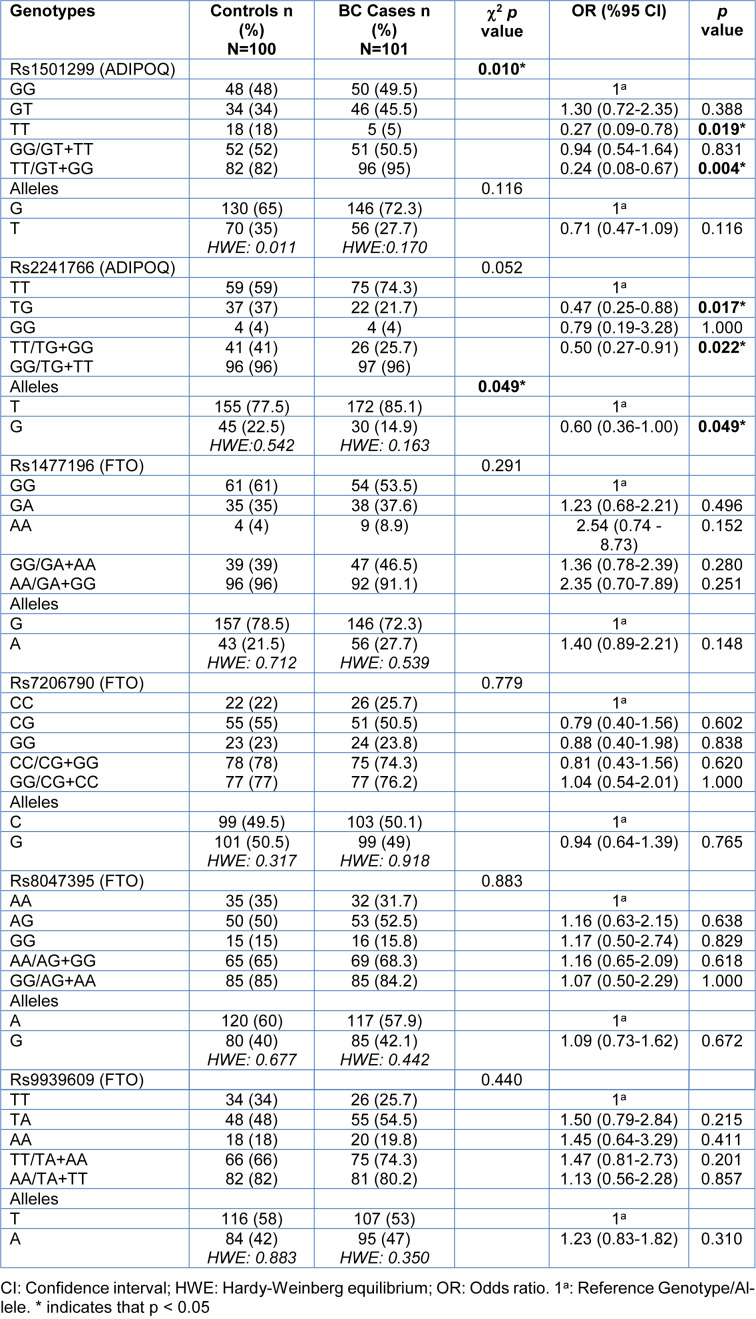
Genotype and allele distributions of ADIPOQ and FTO polymorphisms in BC cases and the control group individuals

**Table 3:**
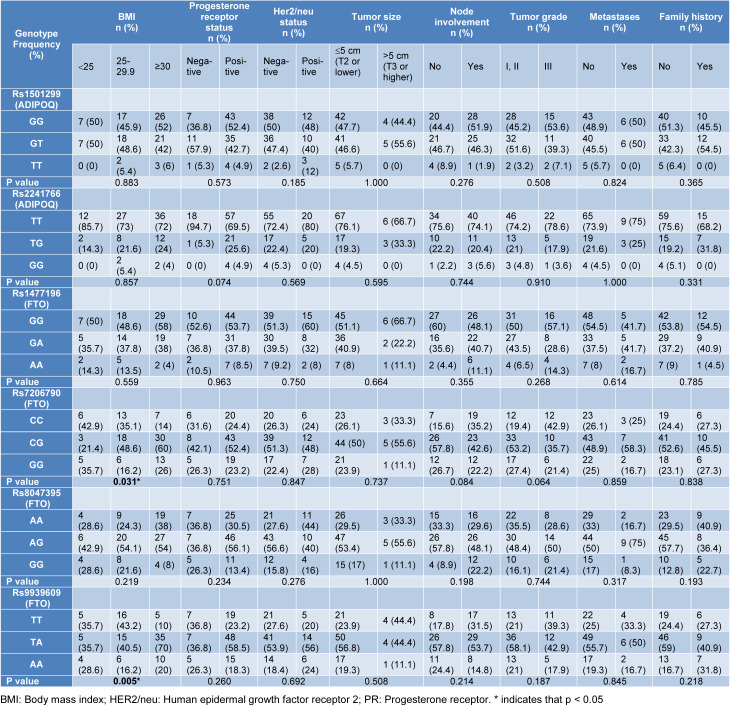
Polymorphisms, histopathological tumor parameters and some characteristics of BC patients
